# Metacognition and self-concept: Elaborating on a construct relation in first-grade children

**DOI:** 10.1371/journal.pone.0250845

**Published:** 2021-04-28

**Authors:** Laura C. Dapp, Claudia M. Roebers

**Affiliations:** Department of Psychology, University of Bern, Bern, Switzerland; University of Oviedo, SPAIN

## Abstract

Self-evaluations play an important role in various fields of study, specifically in research on metacognition and self-concept. Although the assumption that self-evaluations as known from metacognitive monitoring and academic self-concept are related has received wide agreement, the nature of such a relationship has only rarely been investigated. In the current study, the individual-differences approach that has occasionally addressed this association is discussed and extended twofold. For one, a novel way to compare metacognition and self-concept is presented by computing a self-concept bias—analogous to metacognition research. For another, the study targeted a younger population, namely first-grade children. In line with previous studies, the results confirmed a weak relation between metacognitive monitoring and academic self-concept when relating the two constructs at the absolute level of confidence. However, relating the constructs by means of the respective biases revealed a more substantial association. Thus, while previous studies have assumed the common thread between metacognition and self-concept to be best explained by a general confidence trait, the present study suggests the *accuracy* of self-evaluations to be at stake instead. Hence, by introducing a method to quantify a bias in self-concept, the current study proposes a new and promising way to compare and relate the constructs of metacognition and self-concept.

## Introduction

There are several areas of research dealing with an individual’s self-evaluation. For instance, self-evaluations play an essential role in research on metacognition and self-concept. However, stemming from independent research traditions, metacognition and self-concept have always been considered to reflect distinct constructs. Accordingly, they have neither been investigated thoroughly from a joint perspective nor fundamentally linked on a theoretical basis. The present work follows up on one of the few fields of study that have occasionally addressed the relationship between metacognition and self-concept, namely the individual-differences approach that assumes a general confidence trait in individuals. In the following paragraphs, the existing literature is discussed and extended in two different ways. For one, the current study aims to advance the state of research by suggesting a novel and promising way to compare the constructs of metacognition and self-concept. For another, previous research is expanded to younger participants by targeting first-grade children. Thus, while prior studies primarily focused on adults, the present study is exploring the early roots of the relationship between metacognition and self-concept.

Broadly defined, metacognition refers to an individuals’ knowledge about cognition and any cognitive activity that regulates cognitive processes [1]. The regulation of cognition consists of monitoring and control processes, whereby monitoring is defined as the ability to surveil, check, and appraise the quality of one’s own cognitive processes in the course of doing it [2,3]. Confidence judgments—which are in the focus of the present study—constitute an integral part of metacognitive monitoring as they express a person’s belief in the accuracy of a given answer shortly after responding to an item [2]. Hence, confidence judgments reflect self-appraisals related to performance in a specific item (or set of items) of a cognitive task [4].

Self-concept, on the other hand, refers to the knowledge and perception a person has about him- or herself, including self-perceived competence as well as its appraisal in various domains [5,6]. Self-concept is known to be domain-specific, that is, self-concept consists of various subdomains such as social, physical, and academic self-concept [7]. The latter is often subdivided into mathematical and verbal self-concept. In contrast to confidence judgments that usually are—at least theoretically—restricted to the self-perceived performance in one specific item or task, self-concept ratings are based on the self-perceived performance in an entire domain, targeting skills and performance in a broad variety of domain-relevant tasks. Moreover, while confidence judgments directly follow the act of cognitive performance (i.e., providing the answer), self-concept ratings are given in retrospect to a prolonged period of (cognitive) performance. Hence, self-concept can be seen as the broader, overarching, longer-lasting, and probably more abstract construct of self-evaluation [4].

Given the fact that both confidence judgments and academic self-concept reflect the appraisal of one’s own cognitive performance, non-trivial correlations between these constructs have been assumed [4]. However, so far, only little is known about the nature of the relationship between the two constructs [8]. One area of research that has occasionally addressed the relation between metacognitive monitoring and self-concept hails from the individual-differences approach within the metacognition research and has interconnected the two constructs in search of factors explaining overconfidence, a response pattern that is observed in many individuals and particularly pronounced in children. The theoretical background as well as the most relevant findings that emerged from this approach are discussed in the following paragraphs.

Since both confidence judgments and self-concept reflect *beliefs* about one’s own ability or performance, it is not surprising that (similar) deviations from objective performance have been expected to manifest themselves in either research domain. Indeed, a trend toward overconfidence is consistently reported in research from both areas [9–12]. That is, although confidence judgments and self-concept typically are related to performance up to some extent, many individuals tend to have an overoptimistically high sense of how well they are doing. However, while such persistent overconfidence has been of interest in both fields of research, it is—to the best of our knowledge—only in the domain of metacognition that “calibration” has extensively been studied. Within so-called calibration studies, confidence ratings are considered in relation to the objective performance in the respective cognitive test to determine how accurate one’s confidence ratings are. In consequence of this high interest in metacognition accuracy, various measures have been established to quantify the realism in confidence ratings [13, for a review]. In the present study, the focus will be on two common measures accounting for monitoring accuracy, namely resolution and bias. Resolution refers to a discrimination measure that reflects the *relative* accuracy in confidence judgments. The bias, in contrast, reflects the *absolute* accuracy in confidence judgments, and thus, allows for determining the degree of over- or underconfidence, respectively [14,15]. Both resolution and bias have been shown to be well suitable indicators for monitoring accuracy in children [16,17].

Calibration research clearly demonstrates systematic differences between confidence ratings and the accuracy of given answers, indicating an overall bias towards overconfidence in most individuals [10,18]. Certainly, self-perceptions are not equally biased in all individuals, and there is strong evidence for consistent individual differences with respect to the realism in confidence ratings. That is, some individuals are systematically more overconfident than others, and they tend to be very consistent in their overall belief of how well they are doing [10,19–21]. Hence, individuals seem to have an overall sense of self-confidence that is reliable, but not very accurate [10].

As seen from the individual-differences approach, such miscalibration—in most cases overconfidence—is attributed to sources from within the individual [22]. That is, bias in confidence judgments may be a result of a disposition of individuals to appraise their own cognitive work in a certain way [23,24]. Hence, the cause for miscalibration is assumed to lie in a systematic tendency of individuals to express a consistent confidence level that is *irrespective* of their performance [19,25].

In order to explain individual differences within the realism of confidence judgments, researchers have compared (absolute levels of, i.e., noncalibrated) confidence ratings from a broad range of cognitive tests. Indeed—while the correlations between confidence judgments and performance from the same test tend to be significant, indicating substantial realism in confidence ratings [26, for a review]—a considerable amount of research has shown confidence judgments from a broad battery of generic cognitive tests to be consistently intercorrelated as well [23,24,27]. These intercorrelations are high enough to assume a broad and robust self-confidence factor reflecting the habitual way in which individuals appraise the accuracy of their cognitive performance. Given the high stability of confidence judgments, the self-confidence factor has been supposed to feature the properties of a psychological trait, and hence, is often named “confidence trait” [8,28]. There is ample evidence for a general self-confidence trait in adults [4,19,20,23,24,27–29] as well as in children as young as 9 to 12 years [8,30,31]. However, evidence is pending with regard to younger children.

While there is marked evidence for a general self-confidence trait, almost nothing is known about the nature of this trait [32]. If, as seen from the individual-differences approach, self-confidence denotes a psychological trait, then this implies that self-confidence arises from stable person-driven factors in confidence ratings [24,33,34]. Since self-concept—the broad construct of self-evaluation—is known to be stable and sometimes considered a personality characteristic [32,35], a relationship between self-confidence and some well-established areas of self-concept has been assumed [4,27]. More precisely, it has been proposed that self-concept facets that are relevant in cognitive test-taking situations—e.g., the academic self-concept and its sub-facets (i.e., mathematical and verbal self-concept) as well as the memory and the reasoning self-concept—might be related to confidence scores [27,32]. However, the assumed relationship between self-confidence and self-concept has only been studied in a few studies, leaving one with limited empirical support [8,27].

In adults, the general academic self-concept as well as the problem-solving self-concept have been shown to be related to a broad self-confidence trait in a study by Kröner and Biermann [32]. Foremost, Stankov and Crawford [4] reported low, but significant domain-specific correlations between confidence ratings as obtained by the Raven’s Progressive Matrices as well as a vocabulary test with mathematical and verbal self-concept, respectively. Finally, Nietfeld and Schraw [36] showed general mathematical self-efficacy—a proxy for mathematical self-concept—to be related to participants’ self-confidence in a mathematical probability test.

With respect to younger participants, Stankov and colleagues [28] revealed domain-specific associations between confidence and self-concept in 15-year-olds. That is, the English self-concept was related to confidence judgments in an English test, whereas the mathematical self-concept was related to confidence judgments in a mathematics test. Similarly, Efklides and Tsiora [37] found 10- and 11-year-old children’s mathematical self-concept to be related to estimates of solution correctness given after solving mathematical problems. Thereby, the estimates of solution correctness were mainly influenced by the self-concept and, to a lesser degree, by performance, a finding supporting the assumption of confidence ratings being influenced by (or “biased towards”) the self-concept. Finally, a study by Kleitman and Gibson [30] showed the beliefs about one’s own memory and reasoning abilities as well as academic self-efficacy to be related to a broad self-confidence trait in 12-year-olds.

Although these results indicate a weak, yet significant, positive association between metacognitive self-confidence and various measures of self-concept in adults as well as in children, it is important to note that all of them rely on the premise that the measures of self-confidence are composed of *absolute levels* of confidence, that is, that metacognitive self-confidence scores are obtained by averaging the “raw” confidence judgments. Hence, the positive association between self-confidence and self-concept may not be generalizable to any form of *calibrated*, i.e., performance adjusted confidence judgments—which, as stated above, constitute a vital part of metacognition research.

Indeed, some of the studies clearly show the finding of a positive association between self-confidence and self-concept to be limited to the condition where self-confidence is treated on the absolute level, however, that the relationship does not persist when monitoring accuracy is taken into account. For example, in the studies by Nietfeld and Schraw [36] as well as Stankov and Crawford [4], mathematical self-concept was shown to be related to the absolute level of confidence scores, but not to the bias scores. Similarly, verbal self-concept was related to the absolute level of confidence in a vocabulary test, however, once controlling for performance, the relation became insignificant. Taken together, the self-confidence trait seems to be moderately related to various facets of academic self-concept, however, only as long as self-confidence is understood as the absolute level of confidence, i.e., raw confidence judgments. When performance is taken into account—that is, when confidence judgments are calibrated—self-confidence seems to have only little in common with self-concept.

Nonetheless, the realism of individuals’ self-evaluation is of particular interest in both research fields, and the accuracy of confidence judgments literally constitutes a key component in the metacognition research. Confidence ratings per se cannot be regarded as reflecting more accurate confidence judgments. To the contrary, they are akin to expressions of beliefs and, as such, similar to statements known from attitudes scales [4], a fact that may also explain the empirical closeness to self-concept described above. However, having a realistic confidence may constitute a more desirable goal than high certainty per se [38], since accurate self-monitoring is recognized as an essential component for successful learning [18]. Moreover, confidence judgments are not simply the reproduction of a personal disposition of how confident one is about his or her cognitive performance. Rather, they result from an interplay of such general confidence, individual competences, and the actual performance in the applied test [25,32,39]. Hence, it seems purposive to consider the realism in self-evaluations when investigating metacognition—be it as a trait per se or in relation to other constructs.

Although there is no relation between self-confidence and self-concept when confidence ratings are calibrated, it may be premature to conclude that the constructs reflect distinct cognitive processes. When confidence judgments are considered in relation to a measure of performance, but self-concept is not, the two constructs are addressed from different perspectives. In search of the common thread between monitoring and self-concept, however, one should treat the constructs within a unified approach and analyze them as being on a par. Comparing the constructs by relying on absolute levels of self-ratings in both is one way to do so. However, since both monitoring as well as self-concept tend to be positively biased, yet influenced by one’s performance, skills, and achievement, robust insights into the true relationship between metacognition and self-concept may only be obtained by relying on performance-adjusted measures derived from both constructs.

### The present study

The purpose of the current study was to compare children’s self-evaluations in monitoring and academic self-concept by relying on the absolute levels of self-ratings as well as the respective bias scores while building on the existing literature that is, however, dominated by adult data. One of the main objectives was to determine whether the common thread between metacognition and self-concept truly lies in the absolute levels of an individual’s self-evaluation—as assumed in previous studies—or rather in a common disposition towards overconfidence. The former would imply that metacognition and self-concept share an individual’s (trait-like) tendency towards a certain level of self-confidence. The latter, in contrast, would suppose the common aspect to be inherent in the ability to realistically evaluate one’s own cognitive work. To examine the specific nature of the constructs’ overlap, each construct’s absolute level of confidence and bias, respectively, were contrasted. While various measures have been developed to specify the realism in metacognitive monitoring, the accuracy of self-evaluations has not been directly considered in measures of self-concept. By “calibrating” self-concept similar to how it is done with confidence judgments in the metacognition research, the present work presents a novel way to quantify overconfidence in the form of a bias for self-concept. This procedure allows to compare the two constructs on a so far unexplored level, and hence, may provide a deeper understanding of the relationship between metacognition and self-concept.

With respect to the second objective, namely, to expand previous research to younger participants, the present study investigated first-grade children—an age group where self-evaluations related to scholastic performance become highly relevant. In accordance with findings from adult studies, we expected children’s metacognition and self-concept to share a tendency towards a certain level of absolute self-appraisal. Since children tend to be more overconfident than adults, and a process of construct differentiation over the course of development has been reported for other constructs [16], the relation between metacognition and self-concept—with respect to absolute levels—may turn out to be closer in children than in adults. Nevertheless, we expected the constructs’ overlap to be even stronger when relating the constructs by means of the *accuracy* in monitoring and self-concept, respectively. Hence, the current study scrutinizes if a substantial amount of shared processes among metacognition and self-concept in terms of children’s ability to realistically evaluate their own cognitive work can be disclosed.

## Materials and methods

### Procedure

Data came from a study of Swiss first-grade children. The dataset comprised measures of academic achievement (mathematics and literacy), academic self-concept (mathematical and verbal), and metacognitive monitoring (confidence judgments) collected by trained experimenters in children’s schools. While self-concept and monitoring were assessed individually, the testing of academic achievement was done in a class setting. Monitoring was assessed in the context of a learning task and administered on laptops; self-concept and achievement tests were administered in paper-pencil form. The testing session took about 50 minutes per child. The study had been approved by the Faculty of Humanities’ Ethics Committee at the University of Bern, Switzerland, and was realized in accordance with the Declaration of Helsinki. Parents gave written informed consent for their children to participate in the study.

### Participants

One hundred and fifty-five children (71 girls, 84 boys) from the German-speaking part of Switzerland participated in the study. At the time of data collection, children attended first grade and had a mean age of 7 years and 6 months (*SD* = 4.19 months).

### Measures

#### Academic achievement

Academic achievement in mathematics and literacy was assessed using age-appropriate and standardized tests. Three scores each were collected for mathematics and literacy.

*Mathematical achievement*. Mathematical achievement was assessed by three subtests of the “Heidelberger Rechentest” (HRT) [40], a standardized and curriculum-based test of mathematical achievement. These subtests consisted of *magnitude comparison*, continuation of *numerical sequences*, and *addition and subtraction* problems (Cronbach’s α = .80). The reported test-retest reliabilities of the HRT range from *r* = .87 to .93.

*Literacy achievement*. Literacy achievement was assessed by three standardized, curriculum-based tests measuring basic reading and spelling skills. *Reading comprehension* was assessed using the “Salzburger Lese Screening” (SLS) [41] where children had to judge sentences with respect to their meaningfulness. *Speed of reading* was assessed using the “Würzburger Leise Lese Probe” (WLLP) [42] where children had to read a word and identify the matching picture as fast as possible. *Spelling* was assessed by the Hamburger Schreib-Probe (HSP) [43] where children had to write the names of 22 illustrated objects as well as one complete sentence. Cronbach’s α for the three tests accounted for .85. The reported parallel-form reliabilities for the three tests range from *r* = .82 to .98.

#### Metacognitive monitoring

Metacognitive monitoring was assessed in the context of a paired associate learning task [44] where children had to learn the meaning of Japanese symbols, so-called Kanjis. The task consisted of two sequenced sets of 8 Kanjis each. Both sequences were administered in the same order. First, children had to learn the meaning of the Kanjis in a fixed-length encoding phase. Therefore, each Kanji was presented for four seconds together with a color picture showing its meaning. Each trial was preceded by a fixation task where a cross, the attractor, was displayed in the center of the screen for the duration of one second. After the encoding phase, children completed a memory retrieval test where each Kanji was presented in combination with four of the pictures presented before. Children had to select the picture they thought to correspond with the target Kanji. Finally, children were asked how confident they were about their answers by providing confidence judgments for each Kanji on a 5-point Likert-like scale depicted by smiley faces. This smiley scale, introduced to the children before the testing, consisted of five smileys with different smile expressions, ranging from happy to sad. The corresponding verbal labels were *very sure*, *sure*, *neither sure nor unsure*, *unsure*, and *very unsure*. For the analyses, the smileys were numbered from zero (very unsure) to four (very sure). To ensure that children understood the smiley scale, the instruction included a story about a child that had to guess in which of six boxes a ball was hidden. Children understood the use of the smiley scale quickly and solved the three practice questions with ease. For the analyses, measures of absolute level of confidence judgments, monitoring resolution, and bias in confidence judgments were applied.

*Absolute level of confidence judgments*. As a measure for the absolute level of confidence judgments, the mean of all confidence judgments was used (Cronbach’s α = .85). The absolute level of confidence judgments could range from 0 (low confidence) to 4 (high confidence).

*Monitoring resolution*. To determine the *relative* accuracy in monitoring, a discrimination score was computed by subtracting the mean confidence judgment for incorrect trials from the mean confidence judgment for correct trials [14,45–47]. Note that resolution could only be computed for children with at least one error in the memory retrieval test (i.e., 77% of the sample). Monitoring resolution could range from -4 (poor discrimination) to +4 (perfect discrimination).

*Monitoring bias*. The monitoring bias was implemented to determine the *absolute* monitoring accuracy in terms of over- and underconfidence in confidence judgments relative to performance [15,48]. The bias was specified by the absolute discrepancy between the memory retrieval performance (correct or incorrect recognition of the Kanji) and the respective confidence judgment. In case of correct recognition, the confidence judgment “very sure”, constituting the most appropriate confidence judgment, resulted in a discrepancy of zero; the confidence judgment “sure” in a *negative* discrepancy of -1, indicating underestimation of performance; the confidence judgment “neither sure nor unsure” in a discrepancy of -2; the confidence judgment “unsure” in a discrepancy of -3; and the confidence judgment “very unsure” in a discrepancy of -4. In case of incorrect recognition, in contrast, the confidence judgment “very unsure” constituted the most appropriate confidence judgment and resulted in a discrepancy of zero; the confidence judgment “unsure” in a *positive* discrepancy of +1; the confidence judgment “neither sure nor unsure” in a discrepancy of +2; the confidence judgment “sure” in a discrepancy of +3; and the confidence judgment “very sure” in a discrepancy of +4. For every participant, the mean discrepancy across the 16 items was used as an index of monitoring bias. The monitoring bias could range from a performance underestimation of -4 to a performance overestimation of +4, with a score around zero being indicative of accurate self-evaluations.

#### Self-concept

Academic self-concept (mathematical and verbal self-concept; three items each) was assessed by the Pictorial Self-Concept of Attainment Scale (PSCAS) [49,50]. Every item of the PSCAS was designed as a vertical row of 25 stickmen, that is, a realistic size of a child’s class [51]. For every item, children were told that the uppermost stickman represented the best performing and the lowermost stickman the poorest performing child in class in the ability captured by the respective item. The children were instructed to select the stickman that best represented their relative position in class. To ensure that children understood the task correctly, children had to complete a mock item about their relative body height by imaging that their classmates had to line up according to their body height. Children understood the logic and the use of the scale easily. For the analyses, measures of absolute level and bias in academic self-concept were used.

*Absolute level of self-concept*. The absolute level of academic self-concept was computed as the mean of absolute mathematical and verbal self-concept (Cronbach’s α = .79 for all six items). The absolute level of self-concept could range from 1 (low confidence) to 25 (high confidence).

*Self-concept bias*. The bias in academic self-concept was determined by the discrepancy between the domain-specific self-concept (i.e., mathematical and verbal self-concept, respectively) and the academic achievement score in the respective domain. Therefore, the six measures of academic achievement were rescaled to a 25-point scale each, rendering the achievement measures to an equivalent scale length as the six self-concept scales, and hence, providing objective measures of performance to validate the self-concept. Analogously to the procedure described to calculate the monitoring bias, discrepancies between academic achievement and self-concept ratings were computed and then averaged across self-concept domains. The self-concept bias score could range from an underestimation of -24 to an overestimation of +24, with a bias score around zero being indicative of an accurate and realistic self-concept.

## Results

### Descriptive statistics

Descriptive statistics for the various measures of confidence judgments and self-concept are shown in Table 1 (histograms are provided in S2). The mean accuracy of recall in the Kanji-task was 79% (*SD* = .20), corresponding to approximately 13 correctly remembered items on average. Academic achievement—rescaled to a 25-point scale as noted above—accounted for a mean score of *M* = 12.13 (*SD* = 3.56). The correlation between recall accuracy and academic achievement was *r* = .40 (*p* < .001). All correlations are reported in S1 Table.

**Table 1:** Descriptive statistics.

	*M*	*SD*	Min	Max
Confidence judgments				
Absolute level of confidence judgments	3.23	.68	1.19	4.00
Monitoring resolution	-0.13	.97	-3.34	3.87
Monitoring bias	0.20	.79	-1.37	2.69
Academic self-concept				
Absolute level of self-concept	19.63	3.95	5.17	25.00
Self-concept bias	7.50	4.19	-2.31	20.45

### Realism, confidence, and overconfidence

Analyses revealed that children had achieved the ability to objectively evaluate their performance—at least to a fundamental degree. That is, children’s confidence judgments were significantly correlated with their performance in the Kanji task (*r* = .241, *p* < .05). Similarly, self-concept was significantly related to the academic achievement in the respective domain (*r* = .343, *p* < .001 for the mathematical domain; *r* = .385, *p* < .001 for the verbal domain). Together, these results indicate some early realism in children’s self-evaluation.

At the same time, results revealed a significant tendency towards overconfidence. Both the absolute level of confidence judgments and the absolute level of academic self-concept were significantly above the respective scale midpoint; t(154) = 22.692, *p* < .001 for confidence judgments; t(154) = 20.904, *p* < .001 for self-concept. Similarly, monitoring resolution was clearly below the value indicating perfect relative monitoring accuracy. A one-sample T-test confirmed that children’s monitoring resolution significantly differed from perfect monitoring resolution; t(119) = -46.496, *p* < .001. Finally, both the bias in monitoring and the bias in self-concept were above zero, suggesting a tendency towards overconfidence in children’s monitoring and self-concept. A series of one-sample T-tests against the value of perfect absolute accuracy confirmed overly optimistic self-evaluations in both constructs; t(154) = 3.125, *p* < .05 for confidence judgments; t(154) = 22.265, *p* < .001 for self-concept.

To compare the amount of confidence and overconfidence in monitoring and self-concept, respectively, the dissimilar scale ranges of confidence judgment and self-concept measures were taken into account by rescaling the bias and the absolute level of confidence judgments to an equal scale length as the respective self-concept measures. A paired sample T-test showed that the absolute level of confidence judgments and the absolute level of self-concept did not differ significantly; t(154) = -1.885, *p* = .061. In contrast, when comparing monitoring and self-concept bias, a paired sample T-test showed the bias in self-concept to be significantly higher than the bias in monitoring; t(154) = 15.949, *p* < .001. Thus, while the absolute level of confidence seems to manifest itself on a similar level, overconfidence seems to be immanent in both constructs, yet more pronounced in self-concept.

### Three types of relation between monitoring and self-concept

**Fig 1:**
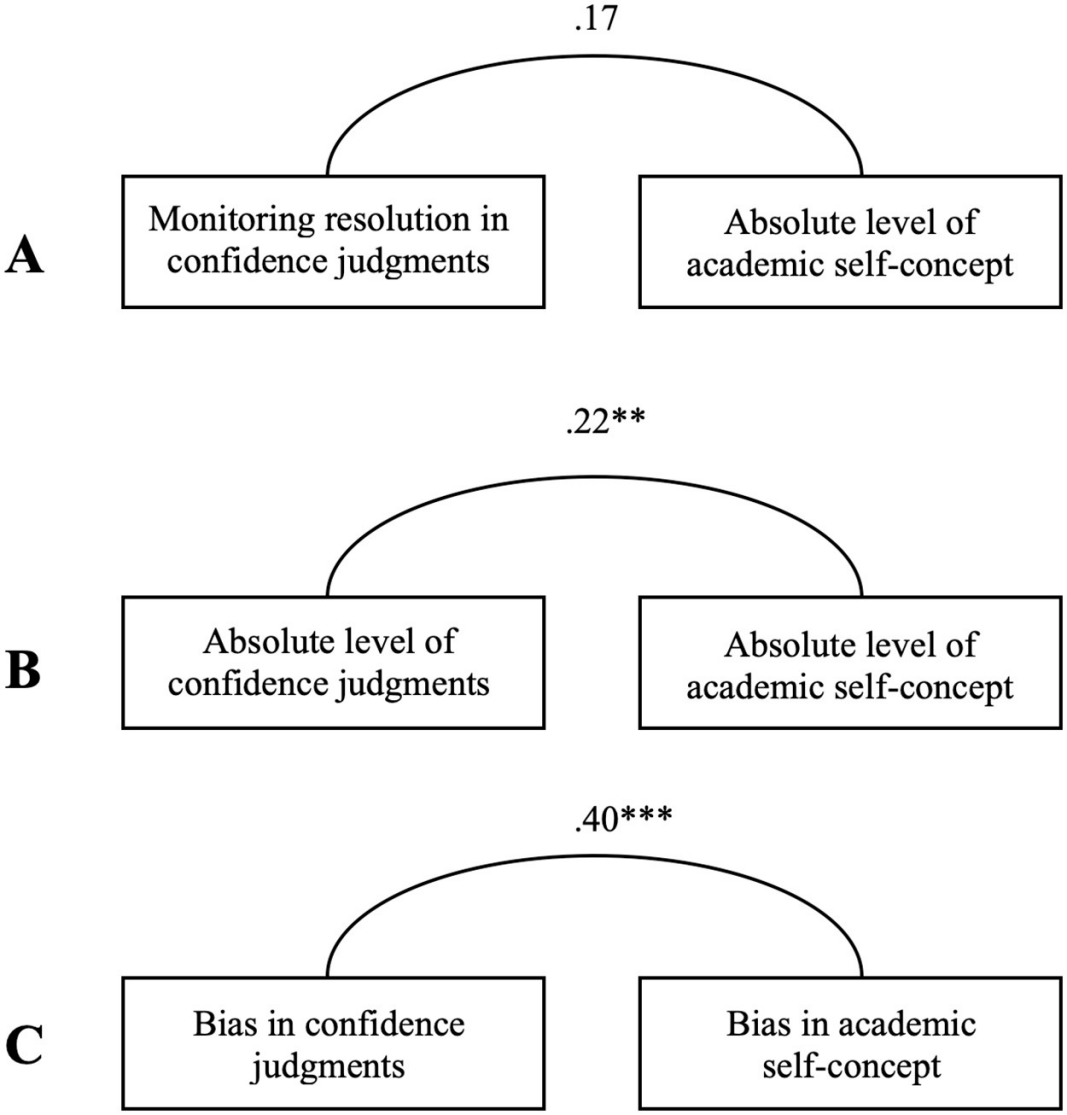
Relations between various measures of confidence judgments and self-concept. **A** = Relation between monitoring resolution and absolute level of academic self-concept; **B** = Relation between absolute level of confidence judgments and absolute level of academic self-concept; **C** = Relation between monitoring bias and academic self-concept bias; ** = *p* < .01; *** = *p* < .001.

To determine the nature of the relations between various measures of monitoring and self-concept, a set of three bivariate correlations was computed. First, monitoring resolution and the absolute level of self-concept, i.e., the state-of-the-art measures in each respective research area, were related. As shown in Fig 1A, resolution and absolute level of self-concept were positively, yet not significantly related to each other. This finding supports the assumption that metacognitive monitoring—in terms of monitoring resolution—and self-concept constitute distinct constructs.

Next, the absolute level of confidence judgments and the absolute level of academic self-concept—two measures reflecting an individual’s subjective perception of success in a specific task or domain, irrespective of the actual performance—were related. As shown in Fig 1B, these absolute levels of self-evaluation were significantly and positively related to each other with a small effect size [52]. Thus, individuals reporting high confidence judgments also tend to have a high academic self-concept.

Finally, the bias in confidence judgments and the bias in academic self-concept were related. Fig 1C displays that these two biases significantly—and even stronger than the absolute levels of self-evaluation—correlated with each other. That is, persons with a smaller bias in their confidence judgments also have a smaller bias in their self-concept.

## Discussion

The present study sought to elaborate on the connection between metacognition and self-concept, two constructs that are based on an individual’s self-evaluation. Although the assumption of a common thread between metacognition and self-concept—such as a common tendency towards biased self-appraisals or a shared cognitive process of self-evaluation—has received wide support on a rather tentative basis, previous research has only sporadically investigated such a relation. Especially with children, the nature of the assumed relationship is far from being fully understood. Moreover, the few studies that aimed at figuring out a common disposition of self-evaluation in metacognition and self-concept primarily focused on absolute levels of self-confidence, and, thereby, largely disregarded the influence of performance. However, since performance, such as monitoring recall accuracy or domain-specific ability, respectively, is expected to be reflected in confidence judgments and self-concept, the present study’s purpose was to extend prior findings by incorporating measures of performance while empirically comparing the constructs of metacognition and self-concept in first-grade children.

Regarding the accuracy of children’s self-evaluations, results showed that children were overconfident in both monitoring and academic self-concept, whereby the bias in self-concept was even more pronounced than the bias in monitoring. Nevertheless, the intercorrelations between the same-test scores revealed the measures of self-evaluation to be significantly related to the measures of performance as well. That is, confidence judgments were significantly related to the recall accuracy in the Kanji task, whereas the mathematical and verbal self-concept were significantly related to the achievement in mathematics and literacy, respectively. Thus, it can be concluded that children’s subjective self-appraisals *do* reflect their objective performance, however, only up to a certain degree, since there was a clear tendency towards overconfidence. Hence, in disentangling the overlap between metacognition and self-concept, it seemed purposive to consider the realism in self-evaluations as well.

In addressing the current study’s primary objective, namely to determine whether the common thread between metacognition and self-concept truly lies in the absolute level of self-evaluations or in a shared ability to provide realistic self-appraisals, three bivariate correlations designed to capture the relation between different measures of self-evaluation were computed. First, the constructs were related by relying on measures reflecting the common state-of-the-art standard in the respective research area, namely relative monitoring accuracy (i.e., resolution) for confidence judgments and the absolute level of self-concept ratings. Next, leaving the self-concept measure unchanged, confidence judgments were considered the absolute level of confidence as well. This approach is consistent with the procedure observed in studies that investigate the general confidence trait. Finally, two analogous measures of absolute accuracy were related, that is, the bias in confidence judgments and the bias in self-concept.

Overall, the results indicate that the relation between children’s metacognitive monitoring and self-concept varies depending on what measures are used in the analyses. When measures of monitoring confidence and self-concept are computed in a way that reflects the state-of-the-art in the respective field of research—that is, when confidence judgments are reflected by a calibration score such as resolution and self-concept by a score of absolute level of self-evaluation—monitoring and self-concept are only very weakly related, a finding confirming the long-standing belief that metacognition and self-concept constitute distinct psychological constructs. In contrast, when the relationship is considered via *the confidence* within both constructs or via *the accuracy of this confidence*, metacognitive monitoring and self-concept show a clear overlap, indicating a common feature of (over-optimistic) self-evaluations. More precisely, comparing the absolute level of confidence in confidence judgments and self-concept revealed a small, yet significant relation between the constructs, whereas comparing the biases in confidence judgments and self-concept revealed an even stronger association. Thus, the common processes of metacognition and self-concept seem to be best addressed by relying on a score reflecting the absolute accuracy of an individual’s self-evaluations.

Regarding the results of detecting a positive relationship between monitoring and self-concept at the absolute level of confidence ratings, yet failing to determine any relationship when accounting for performance in confidence judgments, the present findings are in line with previous research [4,36]. The positive association at the level of absolute ratings suggests an underlying, trait-like disposition of individuals to appraise their own cognitive work in a specific and constant way. Thus, this finding is in agreement with studies describing a general confidence trait [19,23,24,27–29], which has been shown to be weakly related to the broader construct of self-evaluation, namely the self-concept [32]. Notably, the current study indicates an early presence of such a general disposition of individuals towards a constant level of self-appraisal and supports the assumption of self-concept being related to a more general self-confidence trait already in young children.

Since previous research failed to unveil a common thread between metacognition and self-concept once controlling for monitoring performance, up to date, empirical evidence in favor of a common thread was limited to findings based on measures of absolute levels of confidence, that is, measures of self-evaluation that leave the subjects’ performance aside. There are, however, several reasons for why ignoring performance may not be plausible. For one, it is well known that confidence judgments and self-concept *are* related to performance, a finding also confirmed in children [17,50]. Furthermore, the realism of one’s self-evaluation remains to be an important research topic in both fields of study, and calibration is of particular interest in metacognition research. And finally, it should be kept in mind that historically, the general confidence trait literally was explored to explain individual differences in *over* confidence, that is, the deviation between subjective self-evaluation and objective performance. Thus, in search of a common thread regarding metacognition and self-concept, the consideration of performance is inevitable.

In the present study, we extended on previous research by accounting for performance not only with respect to the confidence judgments but also to the self-concept. To the best of our knowledge, we are the first in explicitly “calibrating” the self-concept, that is, providing a measure of discrepancy between self-concept ratings and performance. Hence, having at our disposal a bias score for confidence judgments as well as for self-concept allowed us to compare the constructs on a new and so far unexplored level. In doing so, we could unveil an even stronger link between monitoring and self-concept than by simply relying on absolute levels of confidence. Thus, while monitoring and self-concept indeed share a child’s tendency towards a certain level of self-appraisal, the “true” common thread may rather lie in the ability to *realistically* appraise and evaluate one’s own cognitive performance.

### Limitations and implications

There are some caveats to be considered when interpreting the results. For one, the bias score was significantly more pronounced in self-concept than in monitoring, a finding that may primarily be due to the high mean accuracy in the Kanji task. Since a correct item recognition can only be followed by a realistic or underconfident confidence judgment, the bias in monitoring suffered from a ceiling effect. Moreover, the bias in monitoring was computed based on a larger number of ratings than the bias in self-concept (i.e., 16 items and 6 items, respectively), increasing the probability of a counterbalanced mean bias being closer to zero. Lastly, the two biases were obtained by self-reports measured with different scales, that is, a 5-point smiley scale for the confidence judgments and a 25-point stickman scale for self-concept, whereby the self-concept scale used a relative response format. Accordingly, future studies need to carefully select measurement instruments suitable for countering the mentioned threats. By applying tests resulting in an equally pronounced bias, the link between monitoring and self-concept might be found to be even larger than in the present study.

Further on, it is noteworthy that confidence in monitoring was assessed only with a single task, i.e., the Kanji task. Still, the applied Kanji task can be said to assess children’s cognitive ability on a general level, since it is neither a genuine verbal nor mathematical task. Nevertheless, future studies should account for various sources of confidence judgments to generalize our findings to self-confidence as understood by the broad, general confidence trait. Similarly, we acknowledge that mathematical and verbal self-concept are merely two potential facets of self-concept. While, conceptually, academic self-concept constitutes the most closely related construct, self-appraisals resulting from other self-concept domains may be related to a general confidence trait as well. Thus, an interesting area for future research could be to examine the relation between metacognition and self-concept by adopting a broader perspective on self-concept.

Lastly, the present findings might not be generalizable to the adult population. Investigating and comparing constructs that are based on individuals’ self-evaluation in participants as young as in the present study can be challenging, since children’s ability to introspect and taking themselves as objects of cognitive processing is still in development [16,17,53]. Typically, young children are highly overconfident when evaluating themselves. Thus, the pronounced similarity between monitoring and self-concept when relying on the respective biases, might—at least in part—also be interpreted as mirroring the generally positively biased self-perception of young children [51]. Hence, future research should replicate the disclosed construct relation in adults via multiple measures to corroborate the persistence of the relationship over the course of life.

In sum, the present study opens up new research directions to compare various constructs dealing with an individuals’ self-perception, while more research is needed to confirm and understand the common thread between metacognition and self-concept. Future studies following the presented domain-unifying design would be worthwhile to the broader goal of understanding individual patterns of self-evaluation as well as its development. Finally, future directions might also extend this approach by considering additional psychological constructs related to an individuals’ self-evaluation and cognitive performance.

### Conclusion

In exploring the common thread between metacognition and self-concept, the current study extends the existing research by identifying and comparing a comprehensive set of construct relating measures in elementary school children. Results indicate that it is by and large due to the selected measures whether a substantiated, evidence-based relation between metacognitive monitoring and self-concept can be identified or not; while analyses based on scores well established in the respective research area may result in merely weak, nonsignificant correlations between monitoring and self-concept, analyses adopting a domain-unifying perspective shall unveil a clear overlap of the constructs. The present study suggests that the confluence of self-evaluations from metacognition and self-concept most likely manifests itself when children’s ability to accurately evaluate themselves is concerned. Accordingly, we conclude that the “true” common thread between metacognition and self-concept may best be characterized by an individual’s bias towards overconfidence, hence, the ability for accurate self-evaluations.

## Supporting information

S1 Table Correlation matrix. (PDF)

S1 Fig Histograms for self-evaluation measures. (PDF)

S1 Data (SAV)
