# Supplementary material for: Metacognition and self-concept: Elaborating on a construct relation in first-grade children
Source: PLoS One. 2021 Apr 28;16(4):e0250845. doi: 10.1371/journal.pone.0250845 (PMC8081232; doi:10.1371/journal.pone.0250845)
Supplement: S1 Table — (PDF) [file pone.0250845.s001.pdf]

S1 Table. Correlation matrix

		1	2	3	4	5	6	7	8	9	10
**Performance**											
1	Recall accuracy (Kanji)										
2	Academic achievement[1]	**.400**									
3	Mathematics achievement	**.361**	**.920**								
4	Literacy achievement	**.371**	**.906**	**.668**							
**Metacognition**											
5	Absolute confidence	**.241**	.078	.114	.025						
6	Monitoring resolution	**.400**	.171	.169	.142	-.106					
7	Monitoring bias	**-.746**	**-.309**	-.248	**-.320**	**.416**	**-.188**				
**Self-concept**											
8	Absolute self-concept[2]	.047	**.379**	**.332**	**.361**	**.216**	.168	.146			
9	Self-concept bias	**-.295**	**-.491**	**-.468**	**-.428**	.137	.021	**.400**	**.620**		
10	Mathematics self-concept	.104	**.326**	**.343**	**.249**	**.279**	**.231**	.138	**.881**	**.552**	
11	Verbal self-concept	-.028	**.332**	**.228**	**.385**	.088	.050	.114	**.856**	**.524**	**.509**

Note. Bivariate correlations between all variables. Significant values are shown in bold ($p < .05$).

[1] Academic achievement is the overall variable (used in the manuscript) composed of all items assessing mathematics achievement and literacy achievement.

[2] Absolute self-concept is the overall variable (used in the manuscript) composed of all items assessing mathematics self-concept and verbal self-concept.
