# Supplementary material for: Metacognition and self-concept: Elaborating on a construct relation in first-grade children
Source: PLoS One. 2021 Apr 28;16(4):e0250845. doi: 10.1371/journal.pone.0250845 (PMC8081232; doi:10.1371/journal.pone.0250845)
Supplement: S1 Fig — (PDF) [file pone.0250845.s002.pdf]

S2 Figure. Histograms for self-evaluation measures

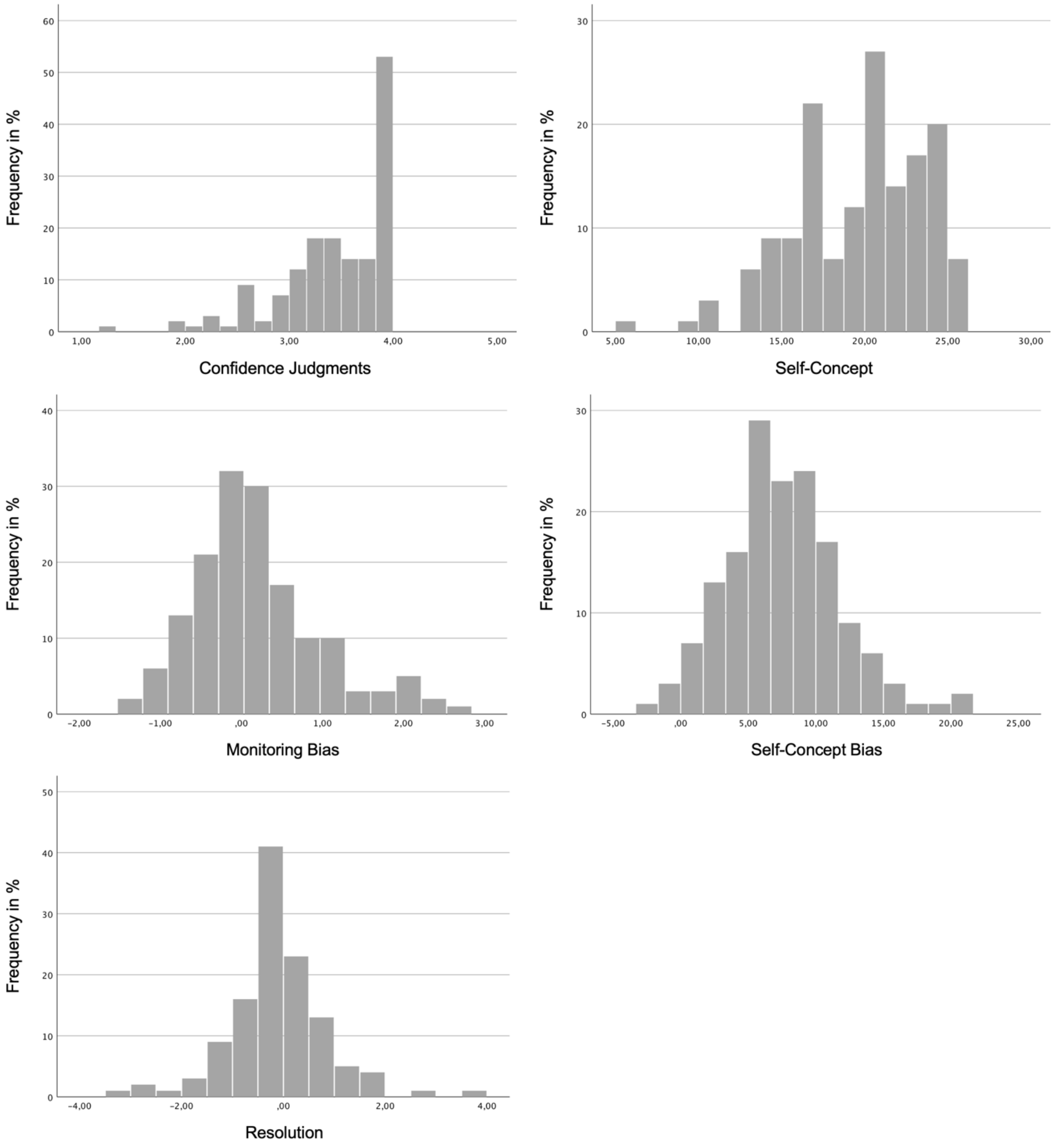

Note. Histograms for analyzed measures of monitoring and self-concept. Confidence judgments and self-concept refer to absolute levels of self-evaluation; monitoring bias and self-concept bias refer to absolute accuracy in self-evaluation; resolution refers to relative accuracy in monitoring.
